# Neoadjuvant nimotuzumab plus chemoradiotherapy compared to neoadjuvant chemoradiotherapy and neoadjuvant chemotherapy for locally advanced esophageal squamous cell carcinoma

**DOI:** 10.18632/oncotarget.23861

**Published:** 2018-01-03

**Authors:** Yongshun Chen, Xiaoyuan Wu, Daxuan Hao, Xinyu Cheng, Lei Zhang, Yougai Zhang, Shaobo Ke, Wei Shi, Chunyu He

**Affiliations:** ^1^ Department of Clinical Oncology, Renmin Hospital of Wuhan University, Wuhan, China; ^2^ Department of Radiation Oncology, Zhengzhou University Affiliated Cancer Hospital, Zhengzhou, China; ^3^ Department of Nephrology, People’s Hospital of Tibet Autonomous Region, Tibet Autonomous Region, Lhasa, China

**Keywords:** esophageal cancer, locally advanced disease, neoadjuvant therapy, EGFR-inhibitor, pathological complete response

## Abstract

Neoadjuvant therapy improves long-term locoregional control and overall survival after surgical resection for esophageal cancer, and neoadjuvant chemotherapy (nCT) or neoadjuvant chemoradiotherapy (nCRT) are commonly used in clinical practice. Nimotuzumab is a humanized monoclonal antibody against epidermal growth factor receptor (EGFR), the efficacy of nimotuzumab added to nCRT for esophageal cancer is uncertain. We conducted this retrospective study in which combining neoadjuvant treatment of nimotuzumab with chemoradiotherapy (Nimo-nCRT) is compared with nCRT and nCT for patients with potentially resectable locally advanced esophageal squamous cell carcinoma. One hundred ninety-five patients received neoadjuvant therapy and 172 (88.2%) underwent esophagectomy. Surgical resection was performed in 94.4% after Nimo-nCRT, versus 92.5% after nCRT and 83.5% after nCT (*P* = 0.026). The R0 resection rate was 100% after Nimo-nCRT, 95.9% after nCRT and 92.6% after nCT (*P* = 0.030). Pathological complete response (pCR) was achieved in 41.2% after Nimo-nCRT, versus 32.4% after nCRT and 14.8% after nCT (*P* = 0.0001). Lymph-node metastases were observed in 29.4% in the Nimo-nCRT group, versus 21.6% in the nCRT group and 35.8% in the nCT group (*P* = 0.093). More patients in the Nimo-nCRT and nCRT group developed grade 3 esophagitis compared to those in the nCT group, *P* = 0.008. There was no difference in surgical complications between the treatment groups. nCRT results in improved R0 resection, higher pCR rate, and a lower frequency of lymph node metastases compared to nCT, adding nimotuzumab to nCRT is safe and appears to facilitate complete resection and increase the pCR rate.

## INTRODUCTION

Esophageal cancer is an aggressively human malignancy, surgical resection by itself provides a high degree of locoregional relapse and distant metastasis for locally advanced disease [1]. Neoadjuvant therapy improves long-term locoregional control and overall survival for esophageal cancer patients undergoing esophagectomy, and two main neoadjuvant approaches are commonly used in clinical practice. The first is neoadjuvant chemotherapy (nCT) using the OEO2 protocol, and demonstrated a 5-year survival improvement of 6% compared to surgery alone [ 1]. The other is neoadjuvant chemoradiotherapy (nCRT) based on the CROSS regimen, which showed a significant improvement in 5-year survival rate in comparison to surgery alone (47% vs. 33%, *P* = 0.003) [2, 3].

R0 resection, pathological complete response (pCR) and downstaging have been regarded as strong and relevant predictors of increased survival in esophageal cancer patients who were undergoing neoadjuvant therapy [1, 4–6], nCRT shows the advantages of effective local therapy in combination with systemic treatment, and the benefits of the radiosensitising effect of chemotherapy compared with nCT. The recently published NeoRES trial in a mixed cohort of 181 patients with esophageal squamous cell carcinoma and adenocarcinoma of the distal esophagus, manifested that nCRT increases the pCR and R0 resection rates and decreases the proportion of patients with metastases in regional lymph nodes compared to nCT, though dose not significantly improve overall survival in squamous cell carcinoma patients [7].

The epidermal growth factor receptor (EGFR) signal pathway plays an important role in the carcinogenesis and progress of esophageal cancer. EGFR expression is observed in 50–70% of esophageal cancer patients and is correlated with inferior prognosis [8, 9]. Nimotuzumab is a recombinant humanized monoclonal IgG1 antibody against human EGFR and it can effectively block the binding of EGF and transforming growth factor-alpha to EGFR. In several phase II studies, nimotuzumab concurrently with chemotherapy and radiotherapy have been proven to be safe and effective in the treatment of esophageal cancer [10–13]. Ramos-Suzarte and colleagues [10] compared nimotuzumab plus concurrent chemoradiotherapy with 5-fluorouracil and cisplatin in the treatment of stage III/IV esophageal squamous cell carcinoma patients and resulted in a great improvement in efficacy (48 vs 15%, *P* = 0.014), the disease control rate (61 vs 27%, *P* = 0.017) and median overall survival (8.1 vs 3.0 months) in the nimotuzumab group. However, the safety and efficacy of the combination of nimotuzumab with neoadjuvant chemoradiotherapy (Nimo-nCRT) in patients with resectable esophageal squamous cell carcinoma is unclear. Therefore, we conducted this study to compare the rate of pCR after Nimo-nCRT with that after nCRT and after nCT. Surgical resection rate, R0 resection rate, downstaging and number of lymph node metastases were also investigated.

## RESULTS

### Patient characteristics

In total, 195 patients with locally advanced squamous cell carcinoma of the thoracic esophagus were included between June 2010 and May 2015. The median age at enrollment was 59 years and the majority of patients were male (*n* = 152, 77.9%). The most common sites of primary tumor were the upper (28.4%) and middle portion (65.1%) of the thoracic esophagus. Preoperative staging showed that 23.6% of patients were clinical stage IIA, 36.4% of patients were stage IIIA, and 33.8% of patients were stage IIIC. Clinical and demographic data for the three groups are shown in Table 1.

**Table 1:** Baseline characteristics at enrollment by treatment group

**Characteristic**	**nCT (*n* = 97)**		**nCRT (*n* = 80)**		**Nimo-nCRT (*n* = 18)**	
	**No.**	**%**	**No.**	**%**	**No**	**%**
Gender						
Male	77	79.4	61	76.2	14	77.8
Female	20	20.6	19	23.8	4	22.2
Age, years						
Median	58		59		61	
Range	33–71		31–74		46–71	
Performance status						
ECOG 0	47	48.5	37	46.2	8	44.4
ECOG 1	50	51.5	43	53.8	10	55.6
Tumor location						
Upper thoracic	20	20.6	29	36.2	6	33.3
Middle thoracic	70	72.2	46	57.5	11	61.1
Lower thoracic	7	7.2	5	6.3	1	5.6
Primary tumor size						
≤ 5 cm	41	42.3	29	36.2	7	38.9
> 5 cm	56	57.7	51	63.8	11	61.1
Clinical stage						
IIA	23	23.7	19	23.8	4	22.2
IIB	7	7.2	2	2.5	1	5.6
IIIA	35	36.1	30	37.5	6	33.3
IIIB	1	1.0	1	1.2	0	0
IIIC	31	32.0	28	35.0	7	38.9

Abbreviations: nCT, neoadjuvant chemotherapy; nCRT, neoadjuvant chemoradiotherapy; Nimo-nCRT, nimotuzumab plus neoadjuvant chemoradiotherapy; ECOG: Eastern Cooperative Oncology Group.

### Toxicity

The most frequently observed hematologic grade 3 or 4 adverse event preoperatively was neutropenia, which was noted in 24.8% of patients in the nCT group, 31.3% in the nCRT group and 27.8% in the Nimo-nCRT group, respectively, no statistical significance was found among the three groups (*P* = 0.640). The incidence of febrile neutropenia was comparable in the three groups (*P* = 0.819). The most frequently occurring nonhematologic grade 3 or 4 adverse events in the three groups were fatigue, anorexia, constipation, nausea, and vomiting.

Compared to those in the nCT group, more patients in the nCRT and in the Nimo-nCRT group developed grade 3 esophagitis (*P* = 0.008). The median time to report of esophagitis was 19 days (range: 15 to 23 months) and 21 days (range: 17 to 24 months) in the nCRT and Nimo-nCRT group, respectively. The toxicities are listed in Table 2. Grade 3–4 radiation pneumonitis was not observed, only one patient in the nCRT group developed Grade 2 radiation pneumonitis by week 2 after treatment. Among the patients in the Nimo-nCRT group, one (5.6%) experienced grade 2 acneiform rash, and four (22.2%) had grade 1 rash. Hypomagnesemia was noted in one patient (5.6%) 4 weeks after the initiation of treatment. The allergic reaction to nimotuzumab was not observed.

**Table 2:** Grade 3 to 4 toxicities associated with the neoadjuvant regimen

**Adverse event**	**nCT (*n* = 97)**		**nCRT (*n* = 80)**		**Nimo-nCRT (*n* = 18)**	
	**Grade 3**	**Grade 4**	**Grade 3**	**Grade 4**	**Grade 3**	**Grade 4**
Hematologic						
Neutropenia	19 (19.6%)	5 (5.2%)	19 (23.8%)	6 (7.5%)	4 (22.2%)	1 (5.6%)
Febrile neutropenia	5 (5.2%)	2 (2.1%)	4 (5.0%)	2 (2.5%)	1 (5.6%)	0
Thrombocytopenia	4 (4.1%)	0	3 (3.8%)	0	1 (5.6%)	0
Nonhematologic						
Esophagitis	3 (3.1%)	0	13 (16.3%)	0	2 (11.1%)	0
Fatigue	8 (8.2%)	0	7 (8.8%)	0	1 (5.6%)	0
Anorexia	12 (12.4%)	2 (2.1%)	9 (11.3%)	1 (1.3%)	2 (11.1%)	0
Constipation	10 (10.3%)	0	7 (8.8%)	0	1 (5.6%)	0
Diarrhea	3 (3.1%)	0	2 (2.5%)	0	0	0
Nausea	22 (22.7%)	0	18 (22.5%)	0	4 (22.2%)	0
Vomiting	10 (10.3%)	0	8 (10.0%)	0	1 (5.6%)	0
Weight loss	2 (2.1%)	0	2 (2.5%)	0	1 (5.6%)	0

Abbreviations: nCT, neoadjuvant chemotherapy; nCRT, neoadjuvant chemoradiotherapy; Nimo-nCRT, nimotuzumab plus neoadjuvant chemoradiotherapy.

Six patients (6.2%) in the nCT group, seven (8.8%) in the nCRT group and one (5.6%) in the Nimo-nCRT group required chemotherapy dose reductions, primarily for neutropenia. Radiotherapy needed to be delayed for six patients (7.5%) in the nCRT group and one (5.6%) in the Nimo-nCRT group, and the treatment interruptions were ranged from 3 to 5 days.

### Surgery

After completion of neoadjuvant therapy, restaging evaluation and the feasibility assessment of performing surgery were conducted by the multidisciplinary team.

Sixteen patients in the nCT group did not undergo esophagectomy, 4 patients developed distant metastases; 11 were deemed surgically unresectable because 10 patients showed stable diseases and 1 had primary tumor progression; one patient refused to undergo surgery. Seventy-five patients (92.6%) underwent an R0 surgical resection of their primary tumor in the 81 patients who proceeded to surgery. The median duration of admission for surgery was 11.5 days (range, 7–58 days), with twelve patients hospitalized for more than 14 days.

Among those in the nCRT group, 74 patients proceeded to surgery and 71 (95.9%) successfully underwent an R0 resection. One patient did not undergo surgery because of progressive disease and four were deemed surgically unresectable, and one patient refused to receive esophagectomy. The median length of hospital stay in those undergoing surgery was 12 days (range 8 to 62 days).

Among the 18 patients in the Nimo-nCRT group, 17 underwent surgery and R0 resections were achieved in all of them. One did not undergo surgery because of patient refusal. The median duration of admission for surgery was 12 days (range, 7–60 days).

**Figure 1:**
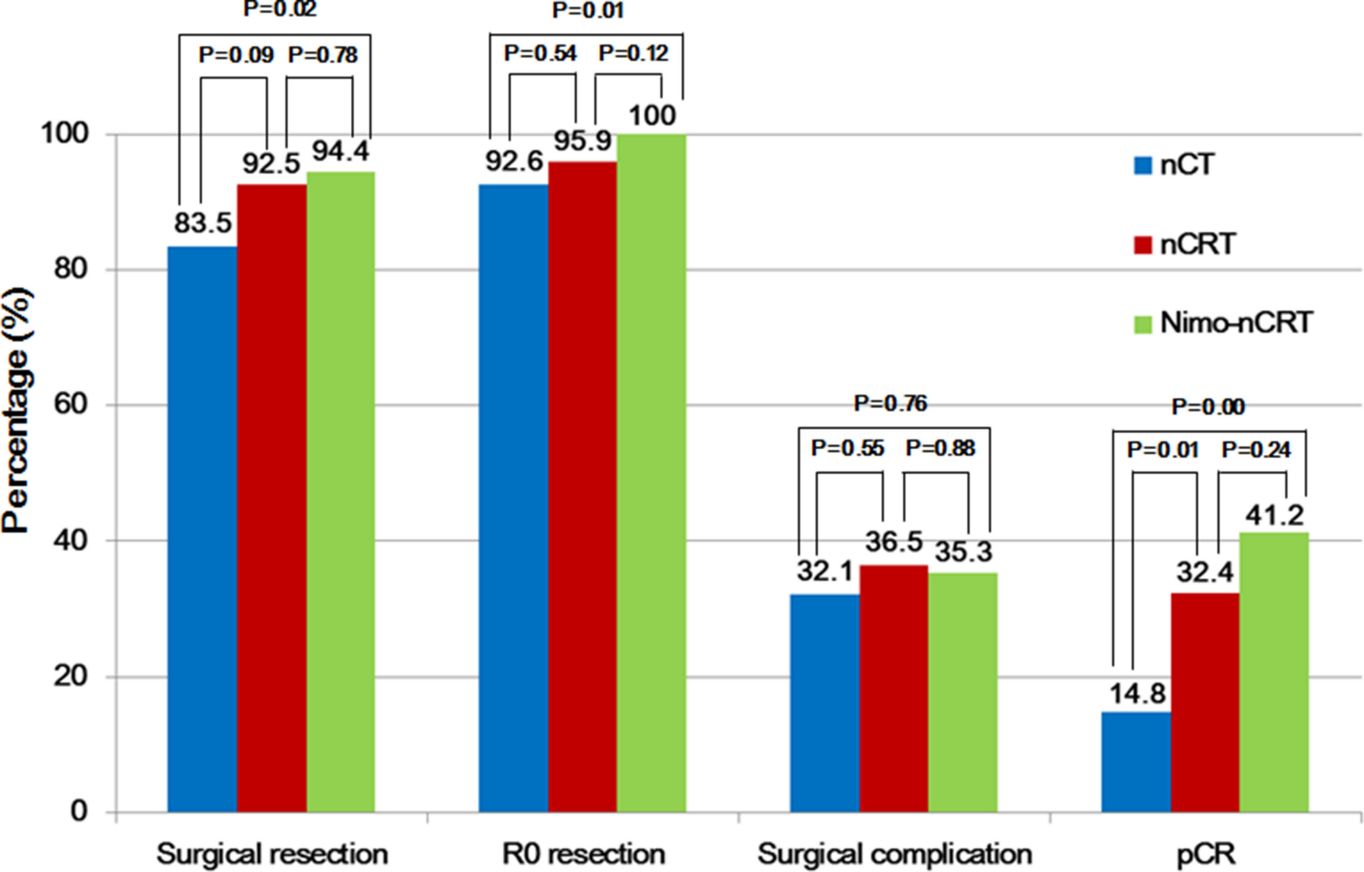
Outcomes related to surgery in the three groups. nCT, neoadjuvant chemotherapy; nCRT, neoadjuvant chemoradiotherapy; Nimo-nCRT, nimotuzumab plus neoadjuvant chemoradiotherapy; pCR, pathologic complete response.

Surgical complications were noted in 32.1% of patients in the nCT group, 36.5% in the nCRT group, and 35.3% in the Nimo-nCRT group, respectively (Figure 1). Infections were the predominant complication of surgery and occurred in about 10% of the patients in the three groups. One patient in the nCT group died within 30 days after surgery because of mediastinal abscese, and one patient in the nCRT group died from respiratory failure on the 52nd day of hospitalization. Other major complications including anastomotic leakage, anastomotic stricture, hoarseness, and arrhythmia were comparable among the three groups (Table 3).

**Table 3:** Postoperative complications

**Complication**	**nCT (*n* = 81)**		**nCRT (*n* = 74)**		**Nimo-nCRT (*n* = 17)**	
	**No**	**%**	**No.**	**%**	**No.**	**%**
Postoperative infection	9	11.1	8	10.8	2	11.8
Anastomotic leakage	4	4.9	4	5.4	1	5.9
Mediastinal abscese	2	2.5	1	1.4	-	-
Anastomotic stricture	4	4.9	5	6.8	1	5.9
Pleural effusion	1	1.2	2	2.7	-	-
Hoarseness	3	3.7	3	4.1	1	5.9
Arrhythmia	3	3.7	4	5.4	1	5.9

Abbreviations: nCT, neoadjuvant chemotherapy; nCRT, neoadjuvant chemoradiotherapy; Nimo-nCRT, nimotuzumab plus neoadjuvant chemoradiotherapy.

### Efficacy

**Figure 2:**
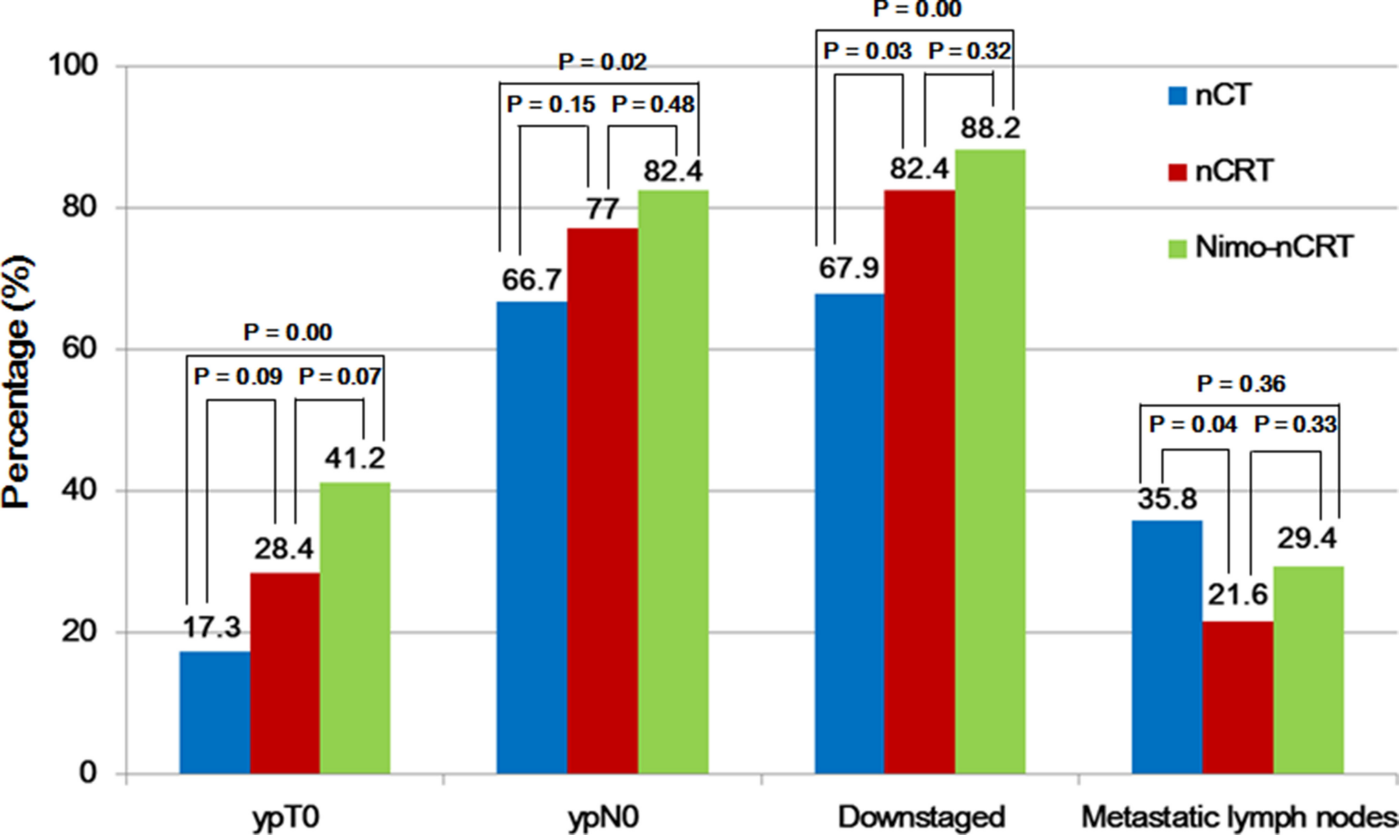
Pathological findings following the three neoadjuvant therapy regimens. nCT, neoadjuvant chemotherapy; nCRT, neoadjuvant chemoradiotherapy; Nimo-nCRT, nimotuzumab plus neoadjuvant chemoradiotherapy; ypT0, pathologic T0 after neoadjuvant therapy; ypN0, pathologic N0 after neoadjuvant therapy.

Pathological findings showed that a pCR was achieved in 41.2% (7/17) of the patients in the Nimo-nCRT group, versus 32.4% (24/74) in the nCRT group and 14.8% (12/81) in the nCT group (*P* = 0.000). Nimo-nCRT was also associated with a significant increased incidence of ypT0 (*P* = 0.001), ypN0 (*P* = 0.043) compared to nCRT and nCT group. Post-operative pathologic staging determined that 88.2% of the patients were downstaged following Nimo-nCRT, versus 82.4% following nCRT and 67.9% following nCT, a significant difference was found among the three groups (*P* = 0.000). Of patients resected in the Nimo-nCRT group, 29.4% had metastatic lymph-nodes, versus 21.6% in the nCRT group and 35.8% in the nCT group (*P* = 0.093; nCRT vs. nCT, *P* = 0.043), as shown in Figure 2.

## DISCUSSION

Neoadjuvant chemotherapy and chemoradiotherapy have convincingly been demonstrated to improve long-term outcomes for patients with locally advanced resectable esophageal cancer, which of the two neoadjuvant therapy types being more beneficial continues to be debated. In 2009, Stahl et al. [14] launched a randomised phase III trial comparing nCRT with nCT; but the study ended prematurely because of slow accrual. One hundred twenty-five patients were randomised, and of whom data of 119 patients were analysed. The R0 resection rate was considerably higher with nCRT (88 %) than nCT (79 %); pCR was significantly higher with nCRT (15.6 vs. 2%, *P* = 0.03) as was the percentage of tumor-free lymph nodes (ypN0, 64.4%vs. 37.7%, *P* = 0.01). Moreover, nCRT demonstrated a positive trend toward improved 3-year overall survival over nCT (47.4 vs. 27.7%), though that was not statistically significant (*P* = 0.07). Klevebro et al. [7] compared the effects of nCT with those of nCRT in 181 esophageal cancer patients. The patients in the nCRT arm received three cycles of platin/5-fluorouracil and 40 Gy of concomitant radiotherapy. The addition of radiotherapy to nCT resulted in better clinical outcomes, the R0 resection rate was 74% after nCT and 87% after nCRT (*P* = 0.04), pCR was much higher with nCRT (28 vs. 9%, *P* = 0.002), and lymph-node metastases were reported in 62% in the nCT group and 35% in the nCRT group (*P* = 0.001); moreover, survival analysis according to tumor histological type showed a trend towards improved survival among patients with squamous cell carcinoma receiving nCRT.

Studies have demonstrated that paclitaxel is an active agent against esophageal cancer, the single agent activity reached approximately 30%, and it has synergistic anticancer activity in combination with cisplatin [15, 16]. Paclitaxel also acts as a radiosensitizer because it causes G2/M phase cell cycle arrest, the most radiosensitive phase of the cell cycle [17]. The current practice of nCRT in many Western countries is utilizing the CROSS trial regimen with chemotherapy by weekly paclitaxel and carboplatin, and concomitant radiotherapy (41.4 Gy/1.8 Gy per fraction). In that trail, 94% of the patients underwent surgery, the rates of R0 resection, pCR, and lymph node metastases were 92%, 29%, and 31%, respectively [2, 3]. The higher rate of pCR and longer overall survival among patients with squamous cell carcinoma were noted compared with those with adenocarcinoma, thus esophageal squamous cell carcinoma patients can benefited more from nCRT. The most recent randomised trial (NEOCTRE5010) [18] used nCRT consisting of 2 cycles of cisplatin and vinorelbine with concurrent radiotherapy (40 Gy in 20 fractions, five fractions per week). That trial recruited 451 patients of whom 224 were randomly allocated to the nCRT group, and 227 to the surgery alone group, nCRT increased R0 resection rate from 91.2% to 98.4% (*P* = 0.002) and a 43.2% pCR rate was achieved.

Neoadjuvant chemoradiotherapy have been proven to prolong the overall survival in patients with resectable thoracic esophageal cancer, but the optimal radiation dose is uncertain, specifically whether low dose CRT is as effective as higher doses. Buckstein and colleagues [19] investigated the neoadjuvant radiation dose and short- and long-term outcomes in the National Cancer Database, 7325 patients with esophageal cancer receiving neoadjuvant CRT followed by curative surgery were identified and four radiation dose levels (40–41.4 Gy, 45 Gy, 50.4 Gy, and 54 Gy) were assessed. After a median follow-up of 26.3 months, radiation dose level was not associated with differences in pCR (*P* = 0.21) or overall survival (*P* = 0.39). The present study used nCRT consisting of two cycles of paclitaxel and cisplatin with concomitant radiotherapy to dose of 40 Gy in 20 fractions, 93% of the patients proceeded to surgery, the rates of R0 resection, pCR, and tumor-free lymph nodes were 96%, 32%, and 78%, respectively. We could conclude from the studies described above, that excellent outcomes can be achieved with low dose radiation and chemotherapy with paclitaxel and cisplatin for neoadjuvant therapy of esophageal cancer.

Anti-EGFR antibodies nimotuzumab and cetuximab inhibit ligand binding to the receptor, thereby stabilize the inactive state of EGFR, nimotuzumab inhibits EGF-stimulated, and ligand-independent signaling in EGFR-overexpressing cells [20]. No data was reported about adding nimotuzumab to concurrent chemoradiotherapy administered preoperatively in patients with locally advanced esophageal cancer, however, several chemoradiotherapy combinations with cetuximab have been reported (Table 4) [21–26]. Kleinberg and colleagues [22] used neoadjuvant oxaliplatin plus fluorouracil and radiotherapy (45 Gy in 25 fractions) in combination with cetuximab, followed by esophagectomy and adjuvant cetuximab and docetaxel, and observed a 32% pCR rate. However, a total of seven deaths occurred during the study’s period because of treatment toxicities and postoperative complications. De Vita F and colleagues [23] treated 41 patients with a regimen consisting of 2 months of cetuximab plus FOLFOX-4 followed by 6 weekly radiotherapy (50.4 Gy in 28 fractions) plus cetuximab. Eight patients achieved pCR among 30 patients who underwent surgery, and the pCR rate was 27%. The most common grade 3/4 toxicity was neutropenia (30%) and skin rash (30%).

Study from Lee et al. [25] and the S0414 trial [27] investigated the toxicity and efficacy of cetuximab in combination with irinotecan, cisplatin, and radiotherapy in patients with locally advanced esophageal cancer. They noted substantial toxicity of high frequency of grade 3 or 4 neutropeniain and grade 3 or 4 diarrhea, but the therapeutic efficacy was low.

**Table 4:** Comparison of studies of chemoradiotherapy regimens with anti-EGFR antibodies

**Year**	**Study**	**Phase**	**Regimen**	**No. of patients (adenocarcinoma: SCC)**	**ITT pCR**
2008	Safran, et al. [21]	II	cetuximab, carboplatin, paclitax, and RT (50.4 Gy/28f)	60 (48:12)	25%
2010	Kleinberg, et al. [22]	II	cetuximab, oxaliplatin, 5-FU, and RT (45 Gy/25f)	22 (22:0)	32%
2011	De Vita F, et al. [23]	II	cetuximab, oxaliplatin, 5-FU, LV, and RT (50.4 Gy/28f)	41 (13:28)	27%
2011	Ruhstaller, et al. [24]	IB/II	cetuximab, cisplatin, docetaxel, and RT (45 Gy/25f)	28 (15:13)	32%
2013	Lee, et al. [25]	II	cetuximab, cisplatin, irinotecan, and RT (50.4 Gy/28f)	19 (16:3)	16%
2016	Lledo, et al. [26]	II	cetuximab, oxaliplatin, 5-FU, LV, and RT (50.4 Gy/30f)	79 (26:53)	NR
2017	This study	II	nimotuzumab, cisplatin, paclitax, and RT (40 Gy/20f)	18 (0:18)	41%

Abbreviations: EGFR, epidermal growth factor receptor; 5-FU, 5-fluorouracil; LV, leucovorin; RT, radiation therapy; SCC, squamous cell carcinoma; ITT, intention to treat; pCR, pathologic complete response; NR, not reported.

Combining cetuximab with a taxane, platinum analog and radiotherapy were well tolerated and had achieved promising results for adjuvant treatment of esophageal cancer. Safran and colleagues [21] treated 60 patients using cetuximab in combination with paclitaxel, carboplatin, and radiotherapy (50.4 Gy in 28 fractions). A 27% pCR rate was achieved in patients who subsequently underwent esophagectomy. The most common grade 3–4 toxicities were rash (25%), dehydration (16%), esophagitis (16%), and neutropenia (14%). Ruhstaller et al. [24] investigated cetuximab added to docetaxel, cisplatin, and radiotherapy (45 Gy in 25 fractions) in 28 esophageal cancer patients who were candidates for potentially curative esophagectomy, 32% of the patients achieved a pCR, and an additional 36% of those had microscopic residual disease. Though these trials presented promising results, the RTOG 0436 trial [28] and the phase III trial of SCOPE1 [29] showed that the addition of cetuximab to standard chemoradiotherapy regimen failed to improve progression-free survival and overall survival irrespective of tumor histology for patients with esophageal cancer suitable for definitive CRT.

Each modality can have adverse effects when esophageal cancer patients are subjected to chemotherapy, biotherapy, radiation, and surgery, safety is thus a major concern of trimodal treatment approach. The studies above demonstrated that the addition of cetuximab increased treatment-related toxicities, decreased the delivery of all components of standard chemoradiotherapy, and might negatively affect treatment outcomes. However, our study showed that the addition of nimotuzumab to neoadjuvant chemoradiotherapy including paclitaxel and cisplatin was safe, no allergic reaction was noted, and skin toxicity was mild. The regimen did not jeopardize the treatment process, and all 17 patients who underwent surgery achieved an R0 resection, the risk of surgical complications was not increased. The rates of pCR and downstaging in patients undergoing Nimo-nCRT were significantly higher than that in those receiving nCRT and nCT.

Our study suggests that adding nimotuzumab to nCRT is safe and may facilitate complete resection and increase the pCR rate for locally advanced esophageal squamous cell carcinoma, the long-term survival and late toxicities are under investigation. The weaknesses of this study are as following: it involves a single-institution experience and the sample size was small, efficacy results should be interpreted with caution; it is a retrospective analysis and the patients, baseline characteristics were not well balance; the biological markers were not evaluated for prognosis. However, our analysis has the following strengths: it focuses on a specific histologic subtype rather than grouping adenocarcinoma and squamous cell carcinoma; it demonstrates that chemoradiation at a dose of 40 Gy using modern radiotherapy technique for neoadjuvant treatment of esophageal cancer is an effective approach.

## MATERIALS AND METHODS

### Patients

Eligible patients had pathologically proven stage II-III thoracic esophageal squamous cell carcinoma (as defined by the American Joint Committee on Cancer [30]). Other eligibility criteria were as follows: aged 18 to 75 years old; Eastern Cooperative Oncology Group (ECOG) performance status score of 0 or 1. Required laboratory parameters for inclusion were hemoglobin ≥ 100g/L, absolute neutrophil count (ANC) ≥ 1.5×10^9^/L, platelets ≥ 100×10^9^/L, ALT or AST < 2.5 times the upper limit of normal (ULN), bilirubin ≤ 1.5 times the ULN, and serum creatinine ≤ 1.5g/L. These participants had no other active malignancy or significant uncontrolled comorbidity, and no prior systemic chemotherapy or chest irradiation.

All patients underwent staging studies including endoscopic ultrasonography (EUS) and EUS guided biopsy; esophagram, neck-to-abdomen computed tomography (CT) scan; or positron emission tomography (PET)/CT scanning, and bronchoscopy if airway infiltration was suspected.

### Chemotherapy

The chemotherapy regimen were administered as follows: paclitaxel given by a 3-hour infusion at a dose of 135 mg/m^2^ on day 1, followed by cisplatin at a dose of 25 mg/m^2^/day on days 1–3. Dexamethasone 20 mg, ranitidine 50 mg and diphenhydramine 50 mg were given intravenously 30 minutes before infusion of paclitaxel. Participants underwent electrocardiogram monitoring for 5 hours after the beginning of the treatment. The chemotherapy was repeated every 3 weeks with two cycles.

### Chemoradiotherapy

The same chemotherapy regimen was administered, and radiotherapy was initiated on the 1st day of the 1st cycle of chemotherapy. Three-dimensional (3D) conformal radiation therapy of 40 Gy (20 fractions of 2.0 Gy) was given once daily for 5 days per week using a high-energy linear accelerator (≥ 6MV). Information from the esophagram, EUS, and CT scan were studied in detail by radiologist and radiation oncologist before delineation of target tumor volume. The gross tumor volume (GTV) included the primary tumor and involved lymph nodes. The clinical target volume (CTV) was created by adding 2cm radially and 4cm cranially and caudally beyond the GTV. The CTV included the medial supraclavicular fossa in patients with tumor located in the upper thoracic esophagus, and CTV also included the celiac nodal region if the primary tumor occurred in the distal third of the thoracic esophagus. The planning target volume (PTV) was generated using an isotropic three-dimensional expansion of the CTV to 6mm. Heterogeneity corrections were performed to assure that at least 95% of the CTV received the prescribed dose.

### Nimotuzumab combined with chemoradiotherapy

The participants in nimotuzumab plus chemoradiotherapy (Nimo-nCRT) group were administrated nimotuzumab by 30 mins I.V. infusion 200 mg weekly for 5 weeks and the same chemoradiotherapy regimen.

### Surgery

Restaging evaluation was performed 4–6 weeks after completion of neoadjuvant therapy, when a neck-to-abdomen CT scan showed no evidence of distant metastasis or inoperable disease, radical esophagectomy with three field lymphadenectomy would be conducted essentially at the sixth week after neoadjuvant therapy.

### Assessments and dose modifications

Toxicity evaluations were measured according to the National Cancer Institute’s Common Toxicity Criteria (Version 1.0) and the RTOG Radiation Morbidity Scoring Criteria. Routine blood tests were carried out once every week, and biochemical liver and kidney functions tests once every two weeks. If a patient experienced allergic reaction to nimotuzumab or paclitaxel, the patients was taken off study. Cisplatin was held for serum creatinine level exceeding 2.0 mg/dL, grade 3–4 ototoxicity, and grade 3–4 neuropathy, and the dose was reduced by half if the creatinine level ranged from 1.6 to 2.0 mg/dL. Paclitaxel was held and subsequently dose reduced by 20% for an ANC < 1000/mm^3^, or platelets < 75,000/mm^3^; for febrile neutropenia or bleeding complications. Radiation was temporarily stopped for any of the following: ANC < 1000/mm^3^, platelets < 50,000/mm^3^, grade 3 esophagitis/mucositis, or any grade 4 toxicity. No dose modifications for radiotherapy were allowed.

### Statistical methods and follow-up

Primarily, the pathologic complete response (pCR) rate, tolerability and postoperative complications of the three regimens were compared. A pCR was defined as the absence of cancer cells in the pathologic examination of surgical specimen, the analysis defined tumor downstaging as a decrease in the stage detected on pathological staging after esophagectomy compared to the stage determined in the pretreatment staging workup. An R0 resection is defined as complete tumor excision with negative histological margins. Pearson’s Chi-squared test for categorical variables and Student’s *t*-test for continuous variables were used to compare patients’ baseline characteristics and outcomes between the groups. All *P* values are two-sided and *P*-values of less than 0.05 were considered to indicate statistical significance. All statistical analyses were performed using SPSS software (version 20.0.1; SPSS Inc., Chicago, IL, USA).

Physical examination, evaluation of ECOG PS, barium swallow, cervical/thoracic/abdominal CT were performed 1 month after the completion of all the therapy. These tests were subsequently performed every 3 months for the first year and every 4 months thereafter. Endoscopic examination was performed if there were new symptoms suggestive of local recurrence.
