# Addendum: Neoadjuvant nimotuzumab plus chemoradiotherapy compared to neoadjuvant chemoradiotherapy and neoadjuvant chemotherapy for locally advanced esophageal squamous cell carcinoma

**DOI:** 10.18632/oncotarget.28320

**Published:** 2022-12-06

**Authors:** Yongshun Chen, Xiaoyuan Wu, Daxuan Hao, Xinyu Cheng, Lei Zhang, Yougai Zhang, Shaobo Ke, Wei Shi, Chunyu He

**Affiliations:** ^1^Department of Clinical Oncology, Renmin Hospital of Wuhan University, Wuhan, China; ^2^Department of Radiation Oncology, Zhengzhou University Affiliated Cancer Hospital, Zhengzhou, China; ^3^Department of Nephrology, People’s Hospital of Tibet Autonomous Region, Tibet Autonomous Region, Lhasa, China

**This article has an addendum:** This observational study was summarized from clinical data, therefore the patients’ informed consents were waived. This study was approved by the Ethics Committee.

Original article: Oncotarget. 2019; 10:4069–4078. 4069-4078. https://doi.org/10.18632/oncotarget.23861

